# Stress Conditions Affect the Immunomodulatory Potential of *Candida albicans* Extracellular Vesicles and Their Impact on Cytokine Release by THP-1 Human Macrophages

**DOI:** 10.3390/ijms242417179

**Published:** 2023-12-06

**Authors:** Kamila Kulig, Katarzyna Bednaruk, Elzbieta Rudolphi-Szydło, Anna Barbasz, Ewelina Wronowska, Olga Barczyk-Woznicka, Elzbieta Karnas, Elzbieta Pyza, Ewa Zuba-Surma, Maria Rapala-Kozik, Justyna Karkowska-Kuleta

**Affiliations:** 1 Department of Comparative Biochemistry and Bioanalytics, Faculty of Biochemistry, Biophysics and Biotechnology, Jagiellonian University, Gronostajowa 7, 30-387 Kraków, Poland; 2 Department of Biochemistry and Biophysics, Institute of Biology, University of the National Education Commission, Podchorazych 2, 30-084 Kraków, Poland; 3 Department of Cell Biology and Imaging, Institute of Zoology and Biomedical Research, Jagiellonian University, Gronostajowa 9, 30-387 Kraków, Poland; 4 Department of Cell Biology, Faculty of Biochemistry, Biophysics and Biotechnology, Jagiellonian University, Gronostajowa 7, 30-387 Kraków, Poland

**Keywords:** infection, fungi, immune system, oxidative stress, inflammation

## Abstract

Human immune cells possess the ability to react complexly and effectively after contact with microbial virulence factors, including those transported in cell-derived structures of nanometer sizes termed extracellular vesicles (EVs). EVs are produced by organisms of all kingdoms, including fungi pathogenic to humans. In this work, the immunomodulatory properties of EVs produced under oxidative stress conditions or at host concentrations of CO_2_ by the fungal pathogen *Candida albicans* were investigated. The interaction of EVs with human pro-monocytes of the U-937 cell line was established, and the most notable effect was attributed to oxidative stress-related EVs. The immunomodulatory potential of tested EVs against human THP-1 macrophages was verified using cytotoxicity assay, ROS-production assay, and the measurement of cytokine production. All fungal EVs tested did not show a significant cytotoxic effect on THP-1 cells, although a slight pro-oxidative impact was indicated for EVs released by *C. albicans* cells grown under oxidative stress. Furthermore, for all tested types of EVs, the pro-inflammatory properties related to increased IL-8 and TNF-α production and decreased IL-10 secretion were demonstrated, with the most significant effect observed for EVs released under oxidative stress conditions.

## 1. Introduction

A multitude of different mechanisms are exploited by human cells to deal with different pathological conditions, such as carcinogenesis, vascular disorders, neurodegeneration, or infections caused by pathogenic microorganisms, including bacteria and fungi [1,2,3,4]. Some of the most important mechanisms available to human immune cells while fighting infectious agents include the generation of oxidative stress by producing reactive oxygen species (ROS) during respiratory burst, as well as the release of pro-inflammatory and anti-inflammatory cytokines. 

*Candida albicans* is one of the most widespread fungal commensals, being pathogenic when the host immunity is compromised and causing both mild superficial candidiases and serious systemic infections. The process of initiation and further development of a candidial infection is administrated with the employment of diverse assortments of virulence factors and mechanisms: (i) secretion of hydrolytic enzymes such as phospholipases, proteinases, lipases; (ii) presentation of adhesive proteins on the pathogen’s cell surface; (iii) release of quorum-sensing molecules involved in the communication between microorganisms in the inhabited niche; (iv) morphological polymorphism; (v) formation of organized, multicellular structures—biofilms—often resistant to standard antifungal treatment and host immunity; (vi) production of extracellular vesicles (EVs) [5,6,7,8,9,10]. EVs are a heterogeneous group of cell-derived membranous structures with nanometer sizes that are naturally released by all types of cells. EVs gather and enclose, with a lipid bilayer, various molecules, mainly proteins, peptides, enzymes, toxins, polysaccharides, lipids, and nucleic acids. Therefore, they can play a crucial role in intercellular and interkingdom communication and have a variety of functions in physiological conditions, as well as in pathological processes [11,12,13,14,15]. 

During the development of infections, the environmental conditions related to host niches, where microorganisms reside, change with varying CO_2_ concentration, redox balance, nutrients limitations, or differences in pressure [16,17]. Such alterations affect the pathogen’s cells, resulting in an adaptation of their response and modification of the arsenal of produced factors used to attack the host and interact with host cells and molecules [18,19]. Oxidative stress, which is related to the process of the oxidative damage of different types of cellular molecules, including proteins, lipids, and nucleic acids [20,21,22,23], is an important part of the response of the innate immune system to pathogens [24,25,26], but at a chronic and excessive scale, it can also lead to severe damage of host cells [27,28,29]. The key molecules playing a protective role during the oxidative stress are enzymes responsible for ROS neutralization, such as superoxide dismutase (SOD), catalase (CAT), glutathione peroxidase (GPx), and peroxiredoxins [27,30]. These enzymes also belong to the repertoire of molecules transported by eukaryotic EVs [31].

In the case of *Candida* fungi, the production of EVs is currently well documented for *C. albicans* [32,33,34,35,36,37] and non-*albicans Candida* species—*C. glabrata*, *C. tropicalis*, *C. parapsilosis* [38,39], *C. auris* [36], and *C. haemulonii* [40]. It has been shown recently that the amount of fungal EVs produced and their molecular composition may differ depending on the growth conditions and the presence of stressful milieu [41,42]. Therefore, we hypothesize that the conditions of oxidative stress or host concentration of CO_2_, under which *C. albicans* EVs are produced, may affect the EVs’ release yield, the properties of the vesicles’ components, and the effect of EVs on the host cells. Since there has been no prior functional characterization of these structures formed under conditions mimicking particular host-related stress, in this work, we verified the potential effect of the host environment on the immunomodulatory properties of EVs produced by *C. albicans*, particularly regarding their subsequent impact on immune cells. 

## 2. Results

**Figure 1:**
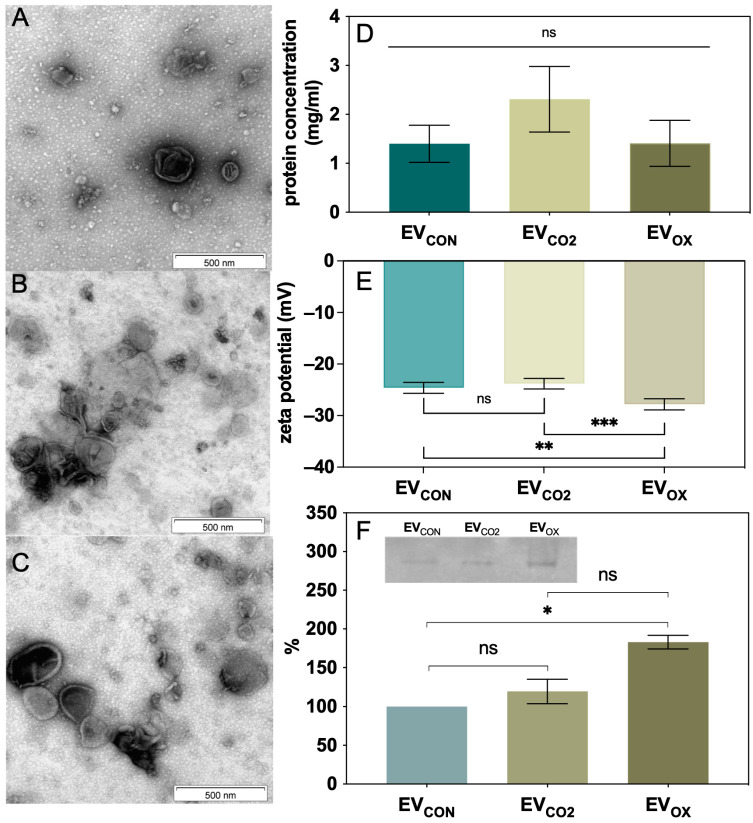
Characteristics of *C. albicans* EVs derived from fungi grown under control conditions, 5% CO_2_, and oxidative stress conditions. TEM image of (**A**) EV_CON_, (**B**) EV_CO2_, and (**C**) EV_OX_. (**D**) The protein content in all three types of EVs. (**E**) The measurement of zeta potential changes measured for EVs. (**F**) Enzymatic activity of EV-derived superoxide dismutase after native gel electrophoresis. The densitometric analysis of electrophoretic gels as a combined result of two independent experiments and the representative gel are presented. The levels of statistical significance are marked with * for *p* < 0.05, ** for *p* < 0.01, *** for *p* < 0.001, and ns when not significant. Scale bar, 500 nm.

The isolation of *Candida* EVs was performed after a 24 h culture of yeast cells on the YPD agar plates under different growth conditions. EVs obtained in the control culture are designated herein as EV_CON_, EVs collected from oxidative stress conditions as EV_OX_, and EVs from *C. albicans* cells cultured in 5% CO_2_ as EV_CO2_. Oxidative stress was induced by the presence of the vitamin K-derived quinone—menadione—leading to the production of intracellular ROS at various cellular locations. Fungal cells were gently scratched from agar plates and suspended in PBS buffer; then, the differential centrifugation technique with the final ultracentrifugation step was applied to eliminate cells and cell debris and subsequently obtain EVs from the supernatant; this method has already been used successfully for the isolation of fungal EVs [42,43,44]. To confirm the presence of EVs in obtained samples, transmission electron microscopy (TEM) imaging was performed, and spherical structures differing in size and surrounded by a lipid bilayer were present in samples from each growth condition (Figure 1A–C). The measured protein concentrations were comparable for all tested samples, with a slight increase for EV_CO2_, although the difference was not statistically significant (Figure 1D). Additionally, the zeta potential measurements for fungal vesicles were conducted with the determined values ranging between −23 and −28 mV (Figure 1E). For EV_OX_, a significant change in the zeta potential was observed compared to EV_CON_ and EV_CO2_, while the latter two did not differ significantly. As the presence of different superoxide dismutases in *C. albicans* EVs was previously reported in proteomic studies [35,41] and as enzymes of this group are essential in the response of *Candida* to menadione-induced stress [45], the SOD-related enzymatic activity was evaluated in each of three types of EVs with the method based on native gel electrophoresis. The obtained results showed the highest SOD activity in EV_OX_. For EV_CON_ and EV_CO2_, the superoxide dismutase activity was also noticeable and rather comparable (Figure 1F). 

**Figure 2:**
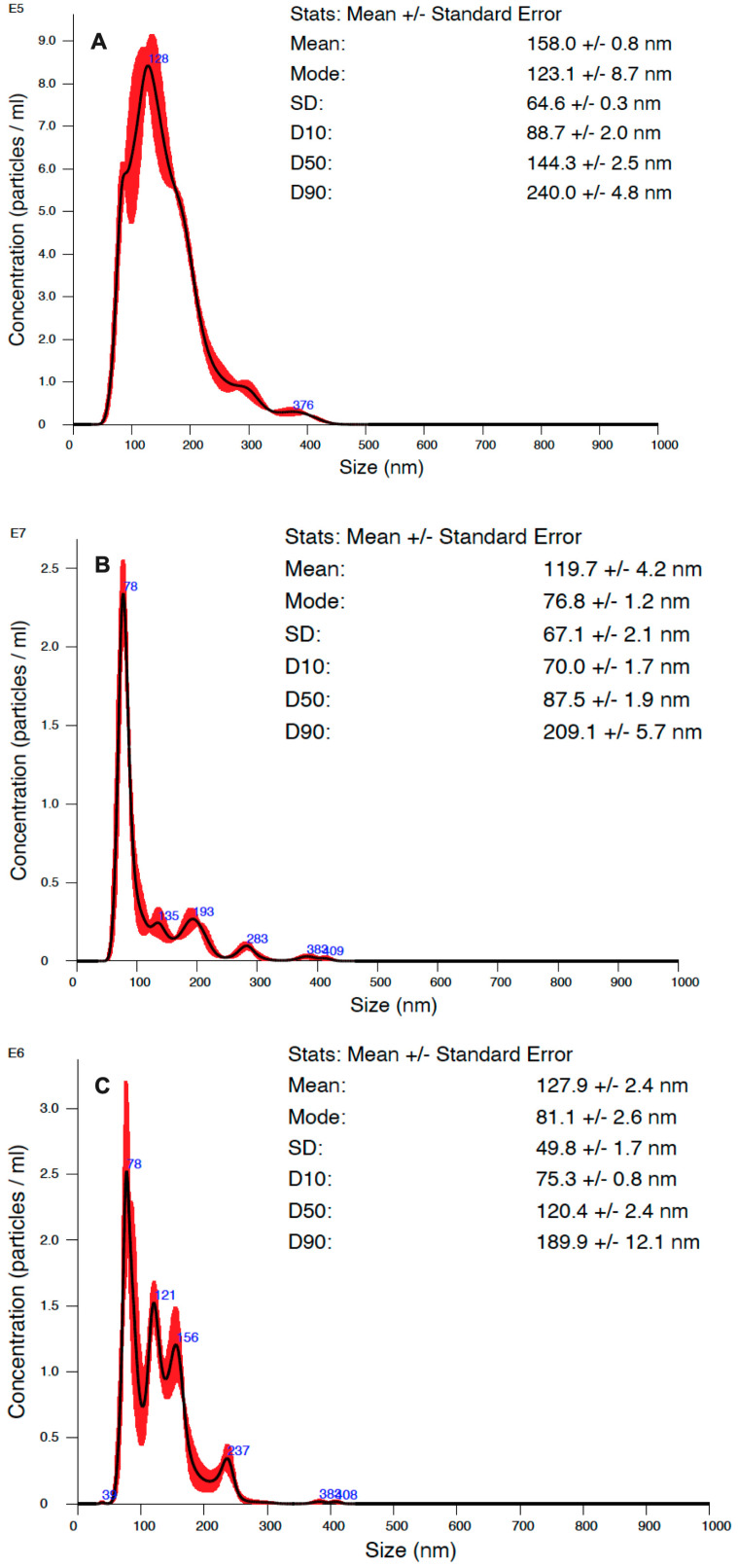
Characteristics of EVs produced by *C. albicans* cells grown under different conditions. NTA particle size distribution analysis of *C. albicans* grown in control conditions (**A**), at host concentration of CO_2_ (**B**), and in oxidative stress (**C**). Representative histograms of the average size distribution from three measurements of a single sample (black line) are presented. The presented numbers indicate the maxima of peaks, and red areas indicate the standard deviation (SD) between measurements. The size parameters of the EVs are included, and factors D10, D50, and D90 mean that 10%, 50%, and 90% of the EV population had a diameter of less than or equal to the presented value.

The measurements with nanoparticle tracking analysis (NTA) were performed to study the size and concentration of *C. albicans* EVs (Figure 2). For *C. albicans* cells grown under the control conditions, the EV_CON_ concentration was 6.87 × 10^10^ EVs/mL, while for EV_CO2_, it was 8.65 × 10^11^ EVs/mL, and for EV_OX_, it was 1.56 × 10^11^ EVs/mL. The sizes of vesicles produced at the host CO_2_ concentration and under conditions of oxidative stress were comparable but considerably lower than those measured for EVs produced under control growth conditions. 

**Figure 3:**
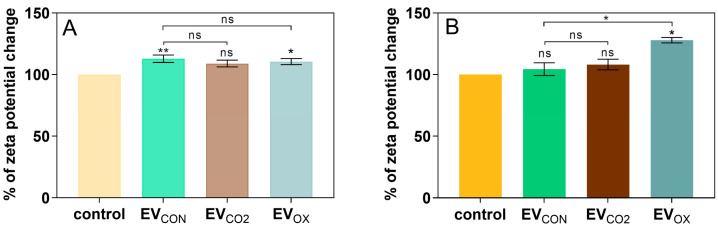
Measurement of changes in zeta potential of (**A**) liposomes with a lipid bilayer modeled after the lipid composition of the cell membrane of U-937 cells and (**B**) U-937 cells as a whole during interactions with *C. albicans* EVs. Cells untreated with EVs served as a control. The levels of statistical significance are marked with * for *p* < 0.05, ** for *p* < 0.01, and ns when not significant (versus control above the bar and between the samples with the line).

To evaluate the impact of growth under stress conditions on the properties related to the ability to interact with human cells, measurements of changes in zeta potential after contact with fungal particles were made for two research models performed in suspension: (i) liposomes, whose membrane modeled the lipid composition of the cell membrane of U-937 cells, and (ii) U-937 cells as a whole. In the first model, for each of the three types of applied EVs, slight changes in the zeta potential of liposomes were observed; however, the most significant changes were noticed for EV_CON_ and EV_OX_ (Figure 3A). In the second model, the zeta potential of U-937 cells during the interaction with EV_CON_ or EV_CO2_ and U-937 cells did not significantly change. In contrast, during interaction between U-937 cells and EV_OX_, the change in the zeta potential of human cells was statistically significant against cells not treated with EVs and cells treated with EV_CON_ (Figure 3B).

**Figure 4:**
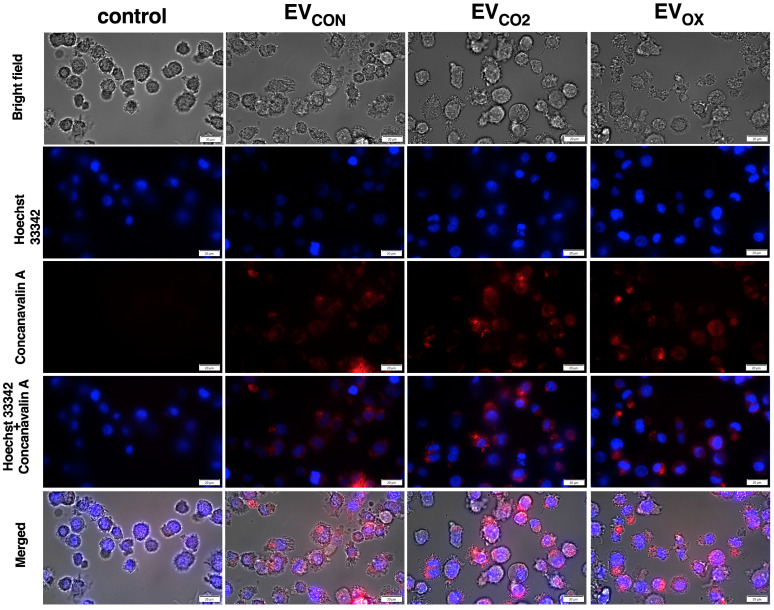
Cellular uptake of *C. albicans* EVs by THP-1 cells. THP-1 cells were incubated for 24 h with fungal EVs labeled with tetramethylrhodamine-conjugated concanavalin A (EVs to cells ratio 10^5^:1) and visualized using an Olympus IX73 microscope. Human cells untreated with fungal EVs, but incubated with fractions collected after mock chromatographic separation of concanavalin A used for staining EVs, served as a control. The cell nucleus was stained with Hoechst 33342. Scale bar, 20 µm. Magnification, 60×.

**Figure 5:**
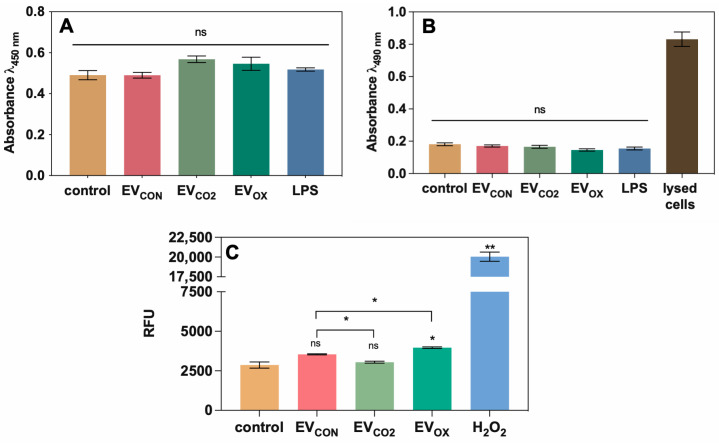
Analysis of (**A**) metabolic activity, (**B**) LDH release, and (**C**) ROS production by THP-1 cells stimulated by *C. albicans* EVs produced by fungi grown under different conditions. Human cells untreated with fungal EVs served as a control. The LPS concentration was 100 ng/mL, (**A**,**B**) and the concentration of H_2_O_2_ was 10 mM (**C**). A representative result of three independent experiments is presented. The levels of statistical significance are marked with * for *p* < 0.05, ** for *p* < 0.01, and ns when not significant (versus control above the bar and between the samples with the line).

**Figure 6:**
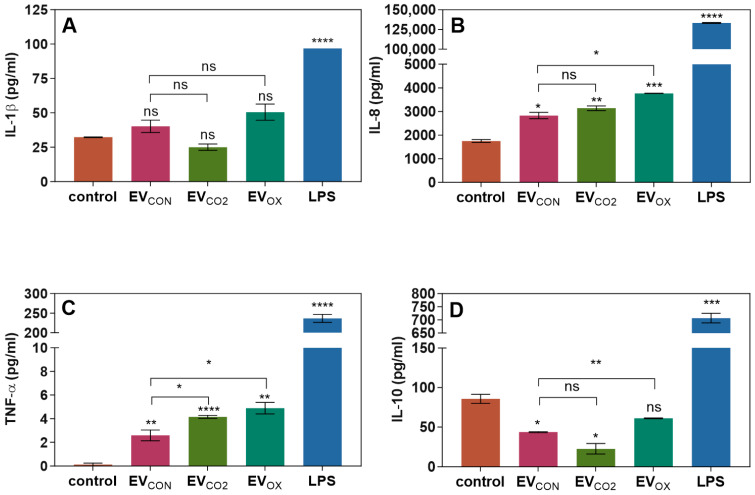
Analysis of the production of cytokines (**A**) IL-1β, (**B**) IL-8, (**C**) TNF-α, and (**D**) IL-10 by THP-1 cells stimulated for 24 h with EVs obtained from *C. albicans* cells grown under different conditions (EVs to cells ratio 10^5^:1). Human cells untreated with fungal EVs served as a control. The concentration of LPS was 100 ng/mL. A representative result of three independent experiments is presented. The levels of statistical significance are marked with * for *p* < 0.05, ** for *p* < 0.01, *** for *p* < 0.001, **** for *p* < 0.0001, and ns when not significant (versus control above the bar and between the samples with the line).

Further functional characteristics of yeast EVs regarding the effect on host cells, including their internalization (Figure 4), possible cytotoxic effect, the stimulation of ROS production (Figure 5), and the immunomodulatory potential (Figure 6), were performed with the human THP-1 cells differentiated into adherent macrophage-like cells. 

A microscopic analysis confirmed that all types of EVs investigated herein interacted with THP-1 cells after 24 h of contact (Figure 4). *C. albicans* EVs were labeled with concanavalin A, as previously described [38], and their location within human cells was visualized using fluorescence imaging with simultaneous staining of the cell nucleus.

The XTT assay did not show significant changes in the metabolic activity of THP-1 cells after their contact with EVs; additionally, the cell damage/LDH release test showed no significant differences between control cells and EV-treated cells (Figure 5A,B). Nevertheless, contact with EV_CO2_ and with EV_OX_ resulted in a slightly increased but statistically insignificant level of cellular metabolic activity (Figure 5A). To measure ROS generation using THP-1 cells, a fluorometric test with dihydrorhodamine 123 was performed (Figure 5C). This test showed that the stimulation of THP-1 cells with all investigated types of EVs resulted in an increase in ROS production compared to untreated cells; the highest and statistically significant increase was observed for EV_OX_. For two other types of EVs, the change in comparison to untreated cells was not statistically significant. As a positive control, H_2_O_2_ in the concentration of 10 mM was used [46]. 

The immunomodulatory potential of fungal EVs was investigated by measuring the concentration of cytokines (IL-1β, IL-8, TNF-α, and IL-10) produced by adherent macrophage-like THP-1 cells and secreted to culture supernatants collected after a 24 h stimulation with *C. albicans* EVs at a final vesicles-to-cell ratio of 10^5^ to 1 (Figure 6). 

The production of IL-1β by human cells was slightly higher after stimulation with EV_CON_ and EV_OX_, while after stimulation with EV_CO2_, the level of IL-1β slightly decreased compared to that of untreated cells. However, the changes were statistically not significant (Figure 6A). Furthermore, all three types of EVs applied stimulated IL-8 production compared to untreated cells, and the effect of EV_OX_ was the greatest compared to EV_CON_ (Figure 6B). Also, the TNF-α production was observed, although at quite low concentrations, and EV_CON_ revealed the lowest immunomodulatory potential, and the highest level of TNF-α production was observed after stimulation with EV_OX_ (Figure 6C). In the case of IL-10 production, in comparison to untreated THP-1 cells, the concentration of IL-10 decreased after stimulation with all types of fungal EVs, however, for EV_OX_ without statistical significance (Figure 6D).

## 3. Discussion

Extracellular vesicles are nanosized structures surrounded by a lipid bilayer and released by both prokaryotic and eukaryotic cells. Those produced by *C. albicans* pathogenic fungi are well characterized in terms of their cargo, as well as some functional properties, as the involvement of candidial EVs in the interactions with human cells and their immunomodulatory properties have been reported repeatedly [32,33,34,35,36,37]. Nonetheless, to the best of our knowledge, the influence of fungal growth under stress conditions on the ability of produced vesicles to affect human cells was shown for the first time in this work, where we tested the impact of host CO_2_ concentration or oxidative stress on EVs activity against human monocytes and macrophages. For fungi, the increased CO_2_ level is an important signal affecting their morphology, nutrient scavenging, drug resistance, formation of biofilm, and virulence [16,47,48]. Furthermore, as one of the important defense mechanisms used by human cells in the response to fungal infection is oxidative burst and the production of ROS causing oxidative damage of microbial cells, the results presented herein may enrich the information about host–pathogen balance during inflammation and chronic diseases associated with the generation of oxidative stress conditions. 

As has been demonstrated so far, in the presence of oxidative stress, the production of EVs by eukaryotic cells can be higher [49], and they may also differ in their molecular cargo and physiological functionality from those produced by cells with a physiological redox status [31]. Consequently, the release of stress-induced EVs can be considered a double-edged sword. They can both play a protective role against oxidative stress for neighboring cells, as such EVs are often enriched in antioxidant enzymes, but they can also transport ROS and oxidized molecules, and thereby contribute to the propagation of oxidative damage [31,50,51]. Furthermore, the situation may be further complicated if it concerns the notable presence of EVs produced by pathogens invading the host organism, which are richly equipped with various virulence factors. Therefore, the state established is related not only to the destructive effects of microbial molecules, but also to the increased activity of immune cells and the robust development of inflammation and oxidative conditions, which, in parallel, can affect pathogen particles. It has been demonstrated previously that EVs released by bacteria and fungi under stress conditions triggered by the presence of antimicrobial drugs differ in terms of size, amount, and immunogenic properties from those produced under controlled conditions, as reported for *Enterococcus faecium* and *Candida auris* [19,52]. Furthermore, previous work by Trentin et al. [42] demonstrated that the presence of menadione as an oxidative-stress inducer could affect the lipid composition of *C. albicans*’ vesicles, and, additionally, the number of EVs produced under these conditions was higher [42]. Therefore, it might be assumed that such stress-related structures may also differ in functionality from those produced under the conditions of undisrupted redox balance. In the proteomic analysis of the content of *C. albicans*’ EVs produced by both fungal yeast-like cells and cells forming biofilms, superoxide dismutases—enzymes playing an important role in neutralizing free radicals—have been identified through mass spectrometry [35,41]. Therefore, the examination of the activity of vesicular SODs was performed in this work. Importantly, it was corroborated herein that SODs located in *C. albicans* EVs are active enzymes. Therefore, it might be assumed that fungal vesicles can provide a specific line of defense against ROS produced by the host, acting at a distance from fungal cells. This activity was detected for all tested vesicles; however, it was particularly noticeable in the case of EVs released by *C. albicans* cells that grow in the presence of oxidative stress. 

Furthermore, the assessment of zeta potential for fungal EVs from various growth conditions also revealed some differences between vesicles. The zeta potential measurements are valuable to assess the surface potential providing information about surface charge stability, being an important parameter when analyzing the interactions between EVs and cells. The initial values or alterations of vesicles’ surface charge are the resultant of charges of biomolecules, being components of EVs and, consequently, may influence the contact and internalization of EVs by human cells [53]. Moreover, environmental conditions affect the zeta potential [54]; therefore, observed differences are informative about changes in the EVs surroundings. Under physiological conditions, the zeta potential of EVs has a negative value, and the analysis presented herein corroborates such observations for EVs produced by *C. albicans* under applied control and stress conditions. EVs derived from other microorganisms also have a negative zeta potential, including EVs produced by protozoan *Naegleria fowleri* with a zeta potential equal to −12.228 mV [55] or *Bifidobacterium longum* and *Lactobacillus plantarum* vesicles with a zeta potential of −11 mV and −27 mV, respectively [56]. The particles with the negative zeta potential are known to be internalized by phagocytic cells, while the particles with a positive charge are known to be internalized by non-phagocytic cells [57]. In this study, the use of two EV-host interaction models—liposomes and U-937 cells—allowed to confirm and further reveal differences in the interactions between control and stress-related fungal EVs and human cell surfaces. Most likely, both the lipids and the proteinaceous components of host structures are potentially involved in the internalization of fungal vesicles. In particular, it could be hypothesized that the involvement of the molecules uniquely present in EV_OX_ in the interactions with surface-exposed host molecules is possibly based on proteins, as suggested by the different interaction of EV_OX_ in comparison to EV_CON_ observed for U-937 cells as a whole, but not for liposomes lacking human surface-displayed proteins.

Subsequently, the interactions of fungal EVs with human macrophage-like cells were investigated using fluorescence microscopy with the use of vesicles labeled with tetramethylrhodamine-conjugated concanavalin A, which specifically bind to vesicular mannoproteins [38]. The internalization of fungal EVs by RAW 264.7 murine macrophage cells has previously been demonstrated for human pathogen *Cryptococcus neoformans* [58] and by murine macrophage-like cells J774A.1 for *Cryptococcus gattii* [59]. Direct interactions of fungal EVs with mammalian macrophages resulted in the modulation of immune cell responses [58,59]. The *C. albicans* EVs examined in this study, which were produced by fungal cells grown under various conditions, were also found to be in direct contact with human macrophages. This was confirmed through microscopic observations, and the fluorescence signals from the labeled EVs were quite consistent across different EV samples. This suggests that regardless of the growth conditions under which they were produced, *C. albicans* EVs have the potential to interact with host cells. 

On the assumption that the effect of host-side stress factors on the composition and functionality of fungal EVs can be considered, it should also be noted that the conditions influencing the activity of microbial EVs may also, in consequence, modify the host response. In response to the pathogen, the host creates a modified environment, which results in the production of altered microbial EVs. Such EVs can in turn affect the host cells differently, thus further modulating their response. Therefore, the phenomenon should be considered bilaterally, and the understanding of the influence of stress conditions generated by host cells during the defensive immune response on the released microbial particles and their cargo is crucial for effective the control of infection [5,8,26,58,59,60,61]. 

In a recent study, the cytotoxic effect of EVs produced by *C. albicans* SC5314 yeast-like cells on THP-1 cells differentiated from macrophage-like cells, measured through LDH activity, was reported as not substantial [37]. Similarly, our study did not show any cytotoxic effects of the three tested types of EVs using the same cell line. Furthermore, the generation of ROS was previously tested for RAW 264.7 cells by stimulating macrophages with *C. haemulonii* cells or EVs, and this resulted in increased ROS production after 24 h of incubation [40]. The viability assay showed an increase in cellular metabolism for some of the used concentrations of *C. haemulonii* EVs, without a cytotoxicity effect on macrophages [40]. Also, the results presented in this work demonstrate that fungal EVs produced by cells under oxidative stress have a slight potential to induce pro-oxidative conditions after contact with host cells, without a significant impact on the viability of the latter. Therefore, this observation proves the involvement of both host and pathogen in the creation of an oxidative environment and in the protection against it.

The immunoregulatory properties of EVs from *Candida* genus have been tested in multiple studies. In the current study, the potential of EVs to influence cytokine release was investigated using THP-1 cells differentiated into macrophage-like cells. The presented results revealed no significant effect of isolated EVs on the IL-1β level; however, the increasing tendency in the production of the chemokine IL-8 was demonstrated for all three types of tested vesicles, while the most significant effect for IL-8 release by THP-1 macrophages was observed for EV_OX_. The levels of TNF-α and IL-10, being pro- and anti-inflammatory cytokines, respectively, suggested rather pro-inflammatory properties of the tested EVs. In the case of TNF-α, both stimulation with EVs produced in the host CO_2_ concentration and in the oxidative stress resulted in the increased production of this cytokine, compared to EVs from control conditions. This may indicate their higher pro-inflammatory potential. In other studies, EVs released by yeast-like forms of *C. albicans* SC5314 were reported as not the stimulating production of TNF-α by THP-1 cells after 24 h of incubation [37]. Additionally, Martinez-López et al. did not reported any significant changes in the levels of IL-10 and IL-12 [37]. In our study, however, a different strain of *C. albicans* was used, and the culture media in which yeasts produced EVs were distinct while the protocols for the differentiation of THP-1 cells into macrophage-like cells were not identical, which may have resulted in the observed differences. In the studies of Zamith-Miranda et al., the analysis of EVs from *C. albicans* and *C. auris* on bone marrow-derived dendritic cells showed an increase in the secretion of IL-6, mainly for *C. albicans* EVs, and a lack of production of other cytokines like TNF-α, IL-10, or IL-12p70 [36]. In other studies, the analysis of EVs produced by *C. albicans* showed an increase in TNF-α, IL-10, and IL-12p40 secreted by RAW 264.7 macrophages, bone marrow-derived murine macrophages, and bone marrow-derived murine dendritic cells [33]. The immunomodulatory effect of fungal EVs was also confirmed for other *Candida* species, *C. glabrata*, *C. parapsilosis*, and *C. tropicalis* in our previous work. After the stimulation of THP-1 cells with EVs, a noticeable increase in TNF-α and IL-8 and the decrease in IL-10 were observed [39]. Furthermore, the functional properties of EVs were tested using an animal model by Vargas et al., in which mice were vaccinated with EVs from *C. albicans* and the analysis performed showed the increased levels of IL-12p70, TNF-a, IL-10, and IL-6 [34]. A similar analysis was also reported for EVs produced by other fungi showing their immunomodulatory function. The stimulation of murine macrophage RAW 264.7 cells with *Cryptococcus neoformans* Evs resulted in the increased levels of TNF-α and IL-10 [58]. The pro-inflammatory effect was also demonstrated for *Paracoccidioides brasiliensis* after incubation with peritoneal macrophage cells, resulting in the increased levels of TNF-α, IL-6, IL-12p40, IL-12p70, IL-1α, and IL-1β and also in the case of the stimulation of macrophage cell line J774A.1, which resulted in greater amounts of released TNF-α, IL-6, and IL-12p40 in comparison to unstimulated cells [62]. The stimulation of macrophages with *Aspergillus fumigatus* EVs showed strongly increased TNF-α levels and a slightly higher amount of IL-10 [63]. Therefore, the stimulation of cytokine production by fungal EVs is a widely recognized phenomenon, but it depends not only on the origin of these particles, but also on the conditions in which they are produced. The presented results provide initial insights into the impact of stress conditions on the effect of EVs produced by *C. albicans* pathogens on host cells. However, for a better understanding of the fungal pathogenesis and host mechanisms of the adaptation to variable environmental conditions and the defensive response during infections and chronic diseases, further research in this area is still needed.

## 4. Materials and Methods

### 4.1. Fungal Strains, Culture Conditions, and EVs Isolation

*Candida albicans* ATCC 10231 yeasts (ATCC, Manassas, VA, USA) were cultured in 20 mL of YPD medium (1% yeast extract, Cat # 70161, 2% soybean peptone, Cat # 70178, and 2% glucose, Cat # G7021; Sigma-Aldrich, St. Louis, MO, USA) for 18 h at 30 °C in an orbital shaker MaxQ 6000 (Thermo Fisher Scientific, Waltham, MA, USA) with rotary speed of 170 rpm. Then, 1 × 10^8^ cells were seeded on YPD agar plates and further cultured for 24 h at 30 °C and ambient air CO_2_ concentration (control culture). For inducing oxidative stress conditions, menadione (Cat # M5625; Sigma-Aldrich) was added [64] at the final concentration of 100 µM to the YPD agar [65,66], and then yeasts were cultured for 24 h at 30 °C and atmospheric CO_2_ concentration. For the culture at host CO_2_ concentration, yeasts seeded on the YPD agar plates were cultured in an atmosphere of 5% CO_2_ and 95% humidity for 24 h at 37 °C. 

Then, the EVs were collected with a protocol described previously [44]. Briefly, yeast cells were scratched from agar plates and transferred to the Eppendorf tube with 1 mL of PBS, pH 7.4 (Cat # L0615; Biowest, Nuaillé, France), and gently stirred with the sterile loop. Then, centrifugation was performed twice, each for 15 min at 4000× *g* at 4 °C to remove the remaining cells and cell debris. The obtained supernatants containing EVs were filtered using an Ultrafree-CL Centrifugal Filter (Cat # UFC40DV25; Merck, Darmstadt, Germany) with a pore size of 0.65 μm to remove cell remnants. The last step was ultracentrifugation for 1 h at 144,000× *g* (*k* factor 112) at 4 °C in polycarbonate thick-wall centrifuge tubes (13 × 64 mm) with 13 mm diameter Delrin tube adapters, using a fixed-angle type 60 Ti Rotor in an Optima™ LE-80K Ultracentrifuge (Beckman Coulter, Brea, CA, USA). Before use, the tubes used for ultracentrifugation and the rotor were sterilized. The collected EVs were transferred in phosphate-buffered saline (PBS; Cat # L0615; Biowest) and filtered through a 0.22 μm filter (Cat # UFC40GV0S; Merck) to Eppendorf tubes and stored at −80 °C for further use. 

### 4.2. Characterization of EVs—NTA Analyses, Protein Concentration Measurements, and TEM Imaging

The nanoparticle tracking analysis (NTA) was applied to estimate the size and concentration of *C. albicans* EVs. A NanoSight NS300 system with camera type sCOS, laser Blue488, and NTA software Version 3.4 (Malvern Instruments, Malvern, UK) was used at 25 °C in a PBS buffer filtered through a 0.22 µm filter (Cat # 17-512F; Lonza, Basel, Switzerland). Each sample was recorded three times for 60 s with a camera level of 13 and a detection threshold parameter of 2. Protein concentration in the EV-containing samples was measured in three biological replicates with *o*-phthalaldehyde (OPA; Cat # 79760; Sigma-Aldrich), and the fluorescence intensity was determined using a Synergy H1 microplate reader (BioTek Instruments, Winooski, VT, USA) with excitation and emission at 340 nm and 455 nm, respectively. Visualization of EVs was performed with a negative-stain transmission electron microscope (TEM) coated with formvar, with 300 mesh copper grids prepared for each EV sample using 2% uranyl acetate (Chemapol, Prague, Czech Republic) as described in a previous work [38,39]. EV imaging was performed using a JEOL JEM-2100 HT transmission electron microscope (JEOL, Tokyo, Japan). 

### 4.3. Labeling of C. albicans EVs with Tetramethylrhodamine-Conjugated Concanavalin A

To prepare fluorescently labeled EVs, 4 × 10^10^ EVs and 10 µg concanavalin A conjugated with tetramethylrhodamine (Cat # C860; Thermo Fisher Scientific) were mixed in 100 µL of PBS, pH 7.4 (Cat # L0615, Biowest), and incubated for 30 min at room temperature in the dark [38]. Then, the sample volume was adjusted to 500 µL with PBS, and separation by size exclusion chromatography was performed with the use of qEVoriginal/70 nm columns (Cat # ICO-70; IZON Science, Christchurch, New Zealand) [67], according to the manufacturer’s instructions, to remove unbound concanavalin A from the sample. The fractions containing EVs were combined and concentrated ten times with 10 kDa-centrifugal filter units (Cat # UFC501024; Merck). A mock chromatographic separation was also completed with a buffer without EVs, containing only concanavalin A-tetramethylrhodamine, to prepare control samples.

### 4.4. Measurements of Superoxide Dismutase Enzymatic Activity

Superoxide dismutase activity was measured with an in-gel activity assay after electrophoretic separation of vesicular proteins [68]. Each of three types of EV samples with a final amount of 200 μg of proteins was lyophilized and resuspended in 10 μL of water. Then, native polyacrylamide-gel electrophoresis (PAGE) was performed, and the obtained gel was stained in the dark for 30 min in the mixture consisting of 50 mM PBS, pH 7.8; 2.43 mM nitrotetrazole blue dye (Cat # N6876; Sigma-Aldrich); 28 mM *N*,*N*,*N′*,*N′*-tetramethylethylenediamine (TEMED; Cat # T-9281; Sigma-Aldrich); 0.14 M riboflavin (Cat # 22038; Reanal, Budapest, Hungary); and 1 mM ethylenediaminetetraacetic acid (EDTA; Cat # E6511; Sigma-Aldrich). After that, the stained gel was exposed to light until reaching visible bands. SOD detection with native PAGE was performed with a set of EVs from two independent biological replicates, and the visualization was completed with the ChemiDoc™ Touch system (Bio-Rad, Hercules, CA, USA). Densitometric analysis was performed using ImageJ software (online version ImageJ.JS) [69].

### 4.5. Cell Culture Conditions

Human pro-monocytic cell line U-937 (human histiocytic lymphoma cell line purchased from ATCC) was cultured in suspension in RPMI 1640 medium (Cat # P04-16500; PAA Laboratories, Cölbe, Germany) containing 5% heat-inactivated fetal bovine serum (FBS) (Cat # P30-19375; Thermo Fisher Scientific) and 0.01% penicillin-streptomycin (Cat # P06-07300; PAA Laboratories). Cells were cultured in the atmosphere of 5% CO_2_ at 37 °C and 85–90% humidity. 

The human monocytic cell line THP-1 (Sigma-Aldrich) was cultured at 37 °C in the atmosphere of 5% CO_2_ and 95% humidity in an RPMI 1640 medium (Cat # L0495; Biowest) supplemented with 10% FBS (Cat # 10270-106; Gibco, Thermo Fisher Scientific). Differentiation from monocytes to adherent macrophage-like cells was performed by treating the cells with 10 ng/mL of phorbol 12-myristate 13-acetate (PMA; Cat # P1585; Sigma-Aldrich) added to a medium with 100 U/mL penicillin and 100 mg/mL streptomycin (Cat # L0022-100; Biowest) for 48 h (with medium exchange after 24 h).

### 4.6. The Zeta Potential Measurements of EVs, Liposomes, and Cells

To characterize fungal EVs, as well as to track their interactions with liposomes modeling the composition of the human cell membrane and with whole human cells, particle electrophoretic mobility in a suspension was determined with the use of a dynamic light scattering technique and Malvern Zetasizer ZS apparatus (Malvern Panalytical Ltd., Malvern, UK) with disposable folded capillary cells (Cat # DTS1070; Malvern). The mobility values were converted to zeta potentials using the Smoluchowski equation. 

Liposomes were created to mimic the membrane of U-937 cells, and the composition of the lipid mixture used included, specifically, 30% 1,2-dipalmitoyl-sn-glycero-3-phosphocholine (DPPC) Cat # 850355C, 24% 1,2-dioleoyl-sn-glycero-3-phosphocholine (DOPC) Cat # 850375P, 13% 1,2-dipalmitoyl-sn-glycero-3-phospho-L-serine (DPPS) Cat # 840037P, 33% 1,2-dioleoyl-sn-glycero-3-phosphoethanolamine (DOPE) Cat # 850725P (Avanti Polar Lipids Inc., Alabaster, AL, USA), and 29% cholesterol (Cat # C8667; Sigma-Aldrich). For liposome preparation, a thin layer of lipids was formed by the evaporation (under argon) of their solution in chloroform wetting the walls of a round-bottom glass tube. The dried film was then dispersed in a defined amount of pure water, and the whole mixture was subjected to ultrasonification and vortexing. To prepare human cells for zeta potential measurements, cells from the U-937 cell line were mixed with the defined physiological 0.9% NaCl solution, and then centrifuged for 5 min at 1000× *g*, and the supernatant was discarded. 

The zeta potentials of the liposomes or human cells in suspension after contact with fungal vesicles were recorded according to the method described in the work of Bondar et al. [70]. Briefly, each of the three types of EVs corresponding to a final protein concentration of 12 μg/mL in PBS, pH 7.4, was added directly to the liposomes or cell suspension (5 × 10^5^ cells/mL). The mixture was incubated for 2 min and the zeta potential was then determined. EVs obtained from three different fungal cultures were administered at least twice to freshly prepared liposomes and cells. Each zeta measurement was repeated at least 5 times.

### 4.7. Stimulation of THP-1 Cells with Fungal EVs

After differentiation of THP-1 cells into adherent macrophage-like cells with 10 ng/mL PMA for 48 h as described above, the growth medium was replaced with a fresh medium without PMA for 3 h, but still with 5% FBS, 100 U/mL penicillin, and 100 mg/mL streptomycin, and then 5 × 10^5^ macrophage-like cells per well in a 24-well microplate were incubated with EVs in the final EVs-to-cells ratio of 10^5^ to 1 in 300 µL for 24 h at 37 °C. Prior to their addition to cells, the EV samples were filtered through a 0.22 µm filter (Cat # UFC30GV0S, Merck).

### 4.8. Analysis of EVs Internalization

The 5 × 10^4^ THP-1 macrophage-like cells, adhered in 96-well glass-like microplate (Cat # P96-1.P; Cellvis, Sunnyvale, CA, USA), were incubated with 5 × 10^9^ EVs bound with concanavalin A-tetramethylrhodamine for 24 h at 37 °C and then washed with 200 µL of PBS and incubated for 5 min with a solution of 1.2 µg of Hoechst 33342 (Cat # 62249; Thermo Fisher Scientific) in 100 µL of PBS. Subsequently, cells and EVs were visualized at 60× magnification with an Olympus IX73 microscope (Olympus, Tokyo, Japan), Hamamatsu Orca Spark camera (Hamamatsu, Hamamatsu City, Japan), and a UPLXAPO60XO lens (Olympus). The images were analyzed using Olympus CellSens Dimension 3.1 software. Representative results from three replicates were selected for presentation.

### 4.9. Cytotoxicity Assay

To determine the metabolic activity of THP-1 cells incubated with EVs, the XTT (sodium 3′-[1-(phenylaminocarbonyl)-3,4-tetrazolium]-bis (4-methoxy6-nitro) benzene sulfonic acid hydrate) (Cat # X6493; Thermo Fisher Scientific) test was performed. Briefly, after 24 h of incubation of cells with EVs as described above, the supernatants were removed for the cytokine assay, and the adhered cells were washed twice with 300 μL PBS (pH 7.4), and then 200 μL of RPMI 1640 medium without phenol red (Cat # L0505; Biowest) and 100 μL of XTT reagent (containing XTT at a final concentration of 1 mg/mL and PMS (*N*-methyl dibenzopyrazine methyl sulphate) at a final concentration of 5 µg/mL (Sigma) were added. Cells were incubated for 1 h at 37 °C in the atmosphere of 5% CO_2_. 

To determine the LDH release after incubation of THP-1 cells with EVs, analysis with Cytotoxicity Detection Kit^PLUS^ (LDH) (Cat # 04744926001; Roche, Basel, Switzerland) was performed according to the manufacturer’s instructions. After 24 h of incubation of macrophage-like cells with EVs, the supernatants were removed, and cells were washed twice with 200 μL PBS. Then, 50 μL of the RPMI 1640 medium without phenol red (Biowest) and 50 μL of a mixed reagent (250 μL of reconstituted catalyst with 11.25 mL dye solution) were added. For positive control, 5 μL of lysis solution was added to the cells. After incubation in the dark for 15 min at 37 °C in the atmosphere of 5% CO_2_, the reaction was stopped by adding 25 μL of stop solution.

After transferring the supernatants to new microplates (Cat # 82.1581; Sarstedt, Nümbrecht, Germany), the absorbance measurement was performed at 450 nm (XTT assay) or 490 nm (LDH assay) using a Synergy H1 microplate reader.

### 4.10. Cytokine Production Assay

After stimulation of THP-1 cells with EVs as described above, supernatants were collected for further analysis of cytokine production, and cell remnants were removed from supernatants by centrifugation (1000 rpm, 5 min). Supernatants collected from unstimulated cells were the negative control, while those from cells stimulated with LPS (Cat # L2630; 100 ng/mL; Sigma-Aldrich) were the positive control. The levels of the production of selected cytokines—IL-1β, IL-8, tumor necrosis factor α (TNF-α) and IL-10—released by macrophage-like cells were measured using Human IL-1β ELISA Set II (Cat # 557953), Human IL-8 ELISA Set (Cat # 555244), Human TNF ELISA Set (Cat # 555212), and Human IL-10 ELISA Set (Cat # 555157) kits (BD OptEIA™), respectively, strictly following the manufacturer’s instructions (BD Biosciences, Franklin Lakes, NJ, USA). 

### 4.11. Intracellular ROS Production

ROS production was evaluated using the DHR test using the measurement of the fluorescence of oxidized dihydrorhodamine 123 (DHR 123) as previously described [71]. The THP-1 cells differentiated to macrophage-like cells, adhering to 96-well glass-like microplate (Cellvis), were stimulated for 2 h at 37 °C with all three types of EVs with a final protein amount of 12 μg in 100 µL RPMI 1640 medium. Cells incubated with the solution of 10 mM H_2_O_2_ (Cat # 885193111; Avantor Performance Materials Poland S.A., Gliwice, Poland) instead of EVs served as a positive control. Then, the THP-1 cells were washed with PBS buffer and incubated with non-fluorescent DHR 123 dye (Cat # D632; Invitrogen, Waltham, MA, USA) at a final concentration of 10 μM for 5 min at 37 °C. The fluorescence intensity of oxidized DHR 123 was measured with excitation at 485 nm and emission at 528 nm using Synergy H1 microplate reader. 

### 4.12. Statistical Analysis

Data are presented as the mean  ±  SD. To analyze the statistical significance, an unpaired *t*-test and a one-way ANOVA with Tukey’s multiple comparisons test were performed with GraphPad Prism software version 9.5.1 (GraphPad Software, La Jolla, CA, USA).

## Data Availability

The data that support the findings of this study are available from the corresponding author upon reasonable request.
